# The Organ of Vision and the Stomatognathic System—Review of Association Studies and Evidence-Based Discussion

**DOI:** 10.3390/brainsci12010014

**Published:** 2021-12-23

**Authors:** Grzegorz Zieliński, Zuzanna Filipiak, Michał Ginszt, Anna Matysik-Woźniak, Robert Rejdak, Piotr Gawda

**Affiliations:** 1 Department of Sports Medicine, Medical University of Lublin, 20-093 Lublin, Poland; piotr.gawda@umlub.pl; 2 Department of Nephrology, Dialysis and Internal Medicine, Medical University of Warsaw, 02-091 Warsaw, Poland; zuzanna.filipiak@interia.pl; 3 Department of Rehabilitation and Physiotherapy, Medical University of Lublin, 20-093 Lublin, Poland; michal.ginszt@umlub.pl; 4 Department of General and Pediatric Ophthalmology, Medical University of Lublin, 20-093 Lublin, Poland; anna.wozniak@umlub.pl (A.M.-W.); robert.rejdak@umlub.pl (R.R.)

**Keywords:** stomatognathic system, organ of vision, neurotology, myopia, TMD, ophthalmology, optometry

## Abstract

The stomatognathic system is a functional complex of tissues and organs located within the oral and craniofacial cavities. The craniofacial anatomical factors and the biomechanics of the temporomandibular joints affect many systems throughout the body, including the organ of vision. However, few scientific reports have shown a relationship between the organ of vision and the stomatognathic system. The purpose of this review is to provide an overview of connections along neural, muscle-fascial, and biochemical pathways between the organ of vision and the stomatognathic system. Based on the literature presented in this review, the connections between the organ of vision and the stomatognathic system seem undeniable. Understanding the anatomical, physiological, and biochemical interrelationships may allow to explain the interactions between the mentioned systems. According to the current knowledge, it is not possible to indicate the main linking pathway; presumably, it may be a combination of several presented pathways. The awareness of this relationship among dentists, ophthalmologists, physiotherapists, and optometrists should increase for the better diagnosis and treatment of patients.

## 1. Introduction

The stomatognathic system is a functional complex of tissues and organs located within the oral and craniofacial cavities [1]. The temporomandibular joint (TMJ) is a paired, functionally coupled joint that was defined by Kaltenborn as anatomically complex, but mechanically simple. Considering the articular disc, it is a modified gliding-hinge joint, according to McConailla’s classification [2]. Okeson described it as one of the most complex joints that build the human body [3]. The TMJ consists of articular surfaces of two bones: temporal and mandibular, which are separated by an articular disc [4]. The masticatory muscle group includes four pairs of muscles: the masseter muscle, the temporal muscle, the medial pterygoid muscle, and the lateral pterygoid muscle [5]. Okeson also points out the important role of the biceps muscle in the masticatory function, and, therefore, according to the above-mentioned author, it can be included in the muscles of the masticatory organ [3]. The superficial temporal and maxillary branches of the external carotid artery supply the TMJ. The auriculotemporal and masseteric branches of the mandibular nerve (V3), which is a branch of the trigeminal nerve, are a sensory supply to the TMJ. [4]. Temporomandibular disorders (TMDs) include issues related to the masticatory muscles, the TMJ, and surrounding tissues [6]. They are primarily characterized by joint and/or muscle pain, acoustic symptoms in the TMJ, and limitation or deviations in the mandibular range of motion [7]. TMDs are recognized by the World Health Organization (WHO) as the third most common dental disease after dental caries and periodontitis. The National Institute of Dental and Craniofacial Research estimates that TMDs affect between 5 and 12% of the population, more often women than men [8]. The ratio of female to male patients with TMDs is 4.1:1 [9]. The annual cost to treat TMDs patients in the United States alone is estimated at 4 billion dollars [8]. The relationship between the parts of this system and the rest of the body, and the impact of its functioning is recognized. For example, the temporomandibular joint is linked to posture, whereas pain from musculotendinous trigger points from the quadriceps region can be transferred to the craniofacial region [10,11,12]. There is a paucity of literature on the effects of vision on dental function [13].

The organ of vision consists of the eyeball, which receives visual impressions; the visual pathways that conduct visual stimuli; and the visual centers in the cortex, where images are processed. Each eyeball is surrounded by seven different bones. The eyeball consists of three membranes: the outer fibrous membrane (sclera and cornea), the middle vascular membrane (iris, ciliary body, choroid), and the inner membrane (retina). The outermost layer is formed by the opaque sclera, which turns into the transparent cornea in the anterior part. Together, they form a rigid, but flexible, structure of the eyeball [14]. The cornea is additionally the main refractive element in the eye. The iris has an opening called the pupil, the width of which varies with light intensity. Behind the cornea, there is an anterior chamber, the posterior border of which is the iris. Rays of light passing through the pupil are refracted by the lens, resulting in an image on the retina. The human eye has two types of photoreceptors: rods and cones. Rods are responsible for night vision, and cones are responsible for day vision. Macula—part of the retina, contains the largest concentration of cones in the eye, which allows us to see small details. Six extraocular muscles are responsible for moving the eyeball in the direction of gaze. The eye artery is responsible for the main vascularization of the eye structures. Seven pairs of cranial nerves are involved in the process of vision. The optic nerve (II) is the most important and is responsible for normal vision. Nerves III, IV, and VI are responsible for innervating the periorbital muscles. The VII nerve is responsible for blinking and the tear reflex. Damage to the VIII nerve can cause rhythmic and uncontrolled eye movements [14]. Myopia is a common condition that develops primarily in childhood and early adulthood, involving the blurring of objects viewed from a distance, and is often the result of abnormal elongation of the eyeball—causing the refractive image formed by the cornea and lens to fall in front of the photoreceptors [15]. Myopia is one of the most common visual problems worldwide, with a prevalence of 10–30% among the adult population in many countries, and up to 80–90% among young adults in the countries of Asia [16,17,18]. It is estimated that by 2050, there will be 4.8 billion myopia patients [19]. Myopia is already the most common cause of irreversible visual impairment in the working population [20,21]. Myopia can be optically corrected with glasses, contact lenses, or refractive surgery. Nevertheless, it is associated with complications, such as cataract, open-angle glaucoma, and retinal detachment [22]. These complications can lead to irreversible visual impairment in later life. Global potential productivity loss in 2015 was estimated at 244 billion dollars associated with uncorrected myopia, and 6 billion dollars due to myopic macular degeneration [20].

The eye, as a sense organ, develops mainly from the neuroepithelium and the ectoderm. The retina develops from the ectoderm, and the lens and cornea, which are the elements that significantly influence the formation of refractive disorders, are formed from the superficial ectoderm. Only muscles, vessels and corneal endothelium and stroma develop from the extracellular mesenchyme. They form an optic vesicle up to the 4th week of embryonic development [23]. On the other hand, soft tissues of the stomatognathic system arise from the mesenchyma at around week 10 of prenatal development [24], and only teeth arise from the ectoderm at around week 5. As such, it would seem that these processes are embryologically separate. However, studies on disorders in the development of the eye and stomatognathic system in diseases such as Down syndrome or ectodermal dysplasia show that gene expression changes and the processes of embryological development of the above structures are delayed [25], which results in a higher percentage of refractive disorders [26], and defects of the stomatognathic system [27].

Based on our recent research, we have also noticed a connection between the organ of vision and the stomatognathic system. Closing and opening the eyes affected the electromyographic patterns of the masticatory and cervical muscles in myopic subjects [28]. There are still no clear scientific reports on the possible connections between the mentioned systems. The aim of this review was to describe connections along neural, myofascial, and biochemical pathways between the organ of vision and the stomatognathic system.

## 2. The Relationship between Organ of Vision and Stomatognathic System Dysfunctions

### 2.1. Visual Stimulation and Masticatory Muscles Activity

The influence of the visual stimulation on masticatory muscle activity has been reported in several studies. The earliest study reached by the authors was Widmalm and Ericsson (1983). They observed that changes in visual stimulation in subjects without visual impairment affected temporal muscle activity and cortical activity (EEG) of patients. Closing both eyes reduces muscle activity by 50–100% [29]. Holmgren et al. (1985) observed that closing the eyes effects a decrease in temporal muscle tension in patients with TMDs [30]. Miralles et al. (1998) detected a significant decrease in sternocleidomastoid and masseter muscle electromyographic activity at rest with eyes closed in a group of healthy subjects, and with myogenic craniocervical dysfunction. However, they observed no change in sEMG activity during maximal voluntary clenching between measurements with open and closed eyes [31]. Monaco et al. (2006) observed that eye closure results in a decrease in temporal muscle activity in people with myopia [32]. Monaco et al. (2006) also observed that corrective lenses in people with visual impairment can improve the bioelectrical tension and balance of masticatory muscles [33]. Spadaro et al. (2010) assessed temporal muscle activity at rest in subjects without visual impairment and TMDs. They showed no differences in sEMG measurements between the open eye and closed eye tests [34]. Ciavarell et al. (2014) evaluated changes in subjects with masticatory muscle pain and myopia relative to healthy subjects in sEMG recordings during an open/closed eye test. In healthy subjects, no differences were shown between the tests. Patients with visual impairment showed large changes between both tests [35]. Fiorucci et al. (2012) reported the absence of changes in temporal muscle activity between open/closed eyes tests in subjects without visual impairment. However, they observed changes in temporal muscle activity between open/closed eyes tests in subjects with visual impairment [36]. Monaco et al. (2020) investigated the effect of standard vision correction on sEMG in TMDs patients compared to subjects without TMDs. The results of their study revealed that TMDs individuals with visual impairment who use standard glasses have considerably higher sEMG values than non-TMDs individuals with visual impairment who use standard glasses. In TMDs subjects, vision correction had no positive effect on stomatognathic or periocranial muscles [37]. Zielinski et al. (2021) observed that closed eyes during sEMG testing were associated with a decrease in activity index values during dental roller clenching, and also a decrease in all functional indices of sternocleidomastoid muscle clenching during clenching in the intermaxillary position. This study also revealed an association with an increase in the functional index of the left temporalis muscle opening compared to testing with eyes open. According to these studies, eye closing and opening in myopic patients may be associated with altered electromyographic patterns in the cervical and masticatory muscles [28].

### 2.2. Structural Disorders in the Stomatognathic System and the Visual Impairment

Cuccia and Cardonna (2008) observed changes in binocular function in patients with temporomandibular disc displacement compared to healthy subjects [38]. Monaco et al. observed that ocular convergence defects were more common in subjects with mandibular lateral deviation than in controls (2004) [39]. They have also suggested a possible association between astigmatism and crossbite (2011) [40] and myopia and Class II division 1 malocclusion (2012) [41]. Bollero et al. (2017) noted that ocular motor defects had a significantly higher prevalence in patients with unilateral crossbite and midline deviation [42]. Vompi et al. (2020) noted a positive association between TMDs and visual impairment. Specifically, associations were found between functional or skeletal orthognathic changes and oculomotor dysfunction. However, the type of relationship could not be determined [43].

## 3. Neurological Connection

### 3.1. Vestibulo–Ocular Reflex Connection

The neurological connection of the stomatognathic and visual systems are focused on the vestibulo–ocular reflex (VOR). Even if the eyes and head are continually moving, as they are in most activities, this reflex keeps the body balanced. Humans’ eye muscles are instantly activated when individuals make a head movement, generating an eye movement that is opposite to the head movement at the same speed. The medial longitudinal fasciculus is responsible for the coordination of movements of the corresponding muscles of the eyeballs. It adjusts the field of view, which, in turn, stabilizes the retinal image, keeping the eye fixed in space, and focused on the object despite head movement [44]. The reflex that additionally supports the achievement of stabilization of the visual target and the image on the retina is the cervico–ocular reflex, which acts in conjunction with the vestibulo–ocular reflex. It is worth noting that information is processed by three sources: the visual system through the eyes, proprioceptive receptors, and vestibular receptors in the inner ear. For the brain to perceive the activity of the vestibular system, it must receive impulses via the afferent pathway from different parts of the vestibular system. The vestibular complex receives afferent information from other parts of the central nervous system, e.g., the reticular formation, parts of midbrain nuclei, cerebellum, and especially from the spinal cord [45].

Proprioceptors are sensory receptors in the inner ear (vestibular receptors), muscles (muscle spindles), tendons (Golgi tendon organ), and joints that inform us about the movement or relative position of our body or body part, responding to stimuli arising internally. Proprioceptors play a key role in vision and visual behavior. Proprioceptive inputs are necessary for oculomotor control, maintenance of binocular vision, and spatial localization [46]. In animals, experimental removal of these inputs led to impaired depth perception, and altered VOR [47,48].

Processes in the brainstem control many of the vestibular reflexes. Connections in the reticular formation, thalamus, and vestibular cortex contribute to body orientation in space and movement awareness. The reticular formation is a complex network of brainstem nuclei and neurons that serve as a major integration and relay center for many important brain systems to coordinate functions necessary for survival. The structure of the reticular formation forms a network-like connection between nuclei and neurons; hence its name “reticular”, which correlates with its function to integrate, coordinate, and influence various regions of the central and peripheral nervous systems. The reticular formation lacks clear boundaries, and the numerous nuclei contained within this structure do not have precise territory delineations, making the reticular formation a difficult structure to characterize and study [49].

The reticular formation receives many direct and indirect afferent stimuli from cutaneous, vestibular, trigeminal, proprioceptive, auditory, and autonomic neurons [45]. The reticular formation also plays an important role in gaze, head movements, and saccadic hand coordination. Different parts of the reticular formation are responsible for different functions of the eye [50]. For example, the paramedian pontine reticular formation coordinates the medial longitudinal fasciculus, which is why it plays a huge role in the coordination of horizontal gaze. The mesencephalic reticular formation coordinates vertical gaze, and the medullary pontine reticular formation coordinates gaze holding and head movements [49]. These regions directly communicate with extraocular motor nuclei, and are crucial for saccadic eye movements. These centers also have connections via descending reticular neurons to coordinate posture and neck movements with eye movements [49].

It is worth noting here that trigeminal neurons project directly to the reticular formation nuclei, and they respond to afferent stimuli through reflex movement. Stimulation of the reticular formation nuclei can lead to increased muscle tone and muscle contractions, e.g., causing facial and neck muscle tremors [51,52]. The sensory nuclei of the trigeminal nerve are so large that they mediate the impulses of all cranial nerves [44].

**Figure 1:**
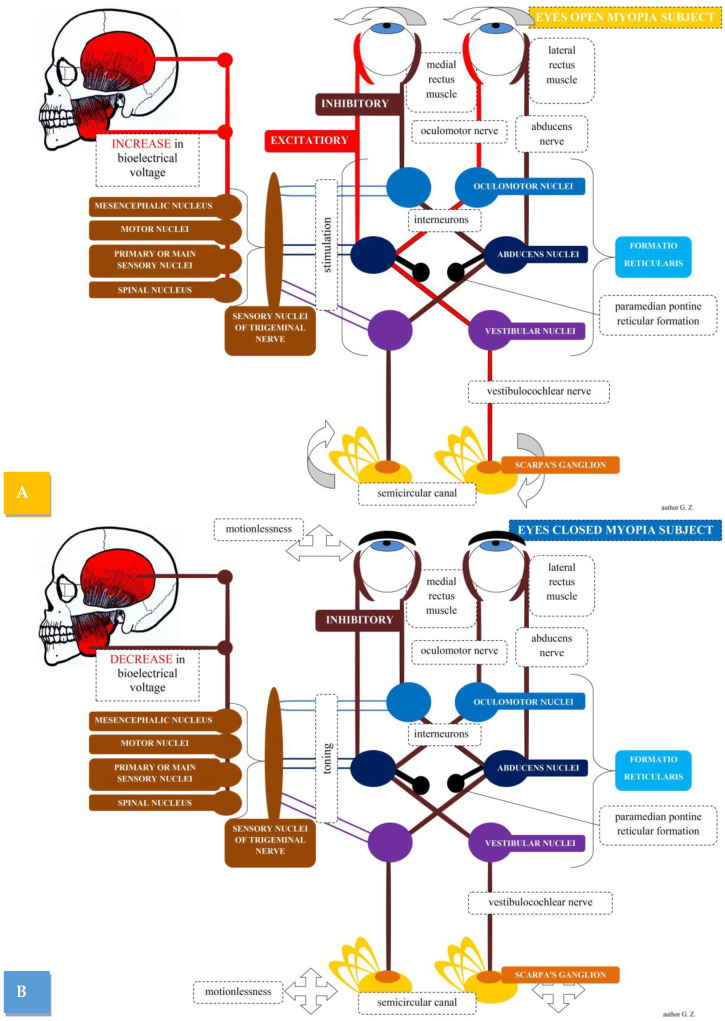
Demonstration of the possible pathway between the muscles of the masticatory organ and the organ of vision in myopic subjects (eyes open—(**A**), and eyes closed—(**B**)) based on the vestibulo–ocular reflex.

The above information may explain the changes in masticatory muscle tone caused by the change in visual stimulus in myopic subjects. The medial vestibular nucleus sends excitatory signals to the contralateral abducens nerve nucleus, and simultaneously sends inhibitory signals to the ipsilateral abducens nerve nucleus. Hypothetically, at this stage, the trigeminal nucleus is stimulated, and sends an impulse to the muscles of the masticatory organ, which causes an increase in activity of the muscles of the masticatory organ, which additionally stabilizes the craniofacial region. Stimulation also goes through the known pathway to the lateral rectus muscle, and through the neurons of the medial longitudinal fasciculus. It transmits excitation to the part of the contralateral oculomotor nucleus innervating the medial rectus muscle. Thus, excitation in the right semicircular canal during head movement causes the eyeballs to turn in the opposite direction (Figure 1).

According to the above-mentioned studies, activity changes occurred mainly in subjects with myopia [32,36], and patients with TMDs [30,35]. Studies in healthy subjects are not consistent regarding changes in activity [29,34].

### 3.2. Central Sensitization Connection

The phenomenon of transferred pain has long been considered one of the possible causes of craniofacial pain; the mechanisms causing this phenomenon have not been explained yet [50]. For example, pain of musculoskeletal origin in people with TMD seems to be related to the presence of muscle-active trigger points and tension, both in the muscles of the masticatory organ, and in the muscles of the cervical spine [53,54,55]. Central sensitization (CS) is a multifaceted spinal–cortical process in which the central nervous system (CNS) amplifies nociceptive sensory stimuli that may then be perceived as experiences of unpleasantness within interneurons originally unrelated to the sensation of that pain, and can cause a central excitatory effect [56]. The concept of CS is based on deregulation of neuronal transmission in the central nervous system that results in hypersensitivity to pain stimuli [56]. The clinical manifestation of the central excitatory effect mechanism depends on the type of interneuron involved. In the case of afferent interneurons, this will be pain transduced, and in the case of efferent interneurons, it will result in increased muscle activity [56]. The cornea is the most densely innervated tissue in the body, with four types of nociceptors innervating its epithelium [46]. Despite their great importance in defending body integrity, the links between nociception, the nervous system specifically encoding potential pain, and vision are still poorly studied [57]. Some studies support a bilateral link between gaze and nociceptive activity. For example, distracting attention from the location of pain results in a slower response to painful stimuli, and a reduction in pain [58]. The study by Filbrich et al. suggests that nociceptive stimuli may affect the perceptual processing of spatially compatible visual inputs [57].

The main hypotheses are related to threat or pain [59]. The phenomenon of transferred pain is caused by afferent impulsion towards the CNS, e.g., in the form of deep pain. It will induce an excitatory effect to nociceptive coupling based on whether visual stimulus in uncorrected myopia will be perceived by the body as nociceptive or not. Another question is whether a change in the visual stimulus in myopic subjects (eyes closed vs. eyes open) would be associated with peripheral silencing of the main nociceptive sites, e.g., the cervical segment, and stomatognathic system. Responses from the stomatognathic system and cervical segment will be transmitted as a response to the nociceptive stimulus to effectors located within the eyeball. The appropriate response to the nociceptive stimulus would be closing the eyes. Closing the eyes hypothetically will decrease activity in the muscles of the pain-affected regions, which are the stomatognathic system and the cervical segment.

**Figure 2:**
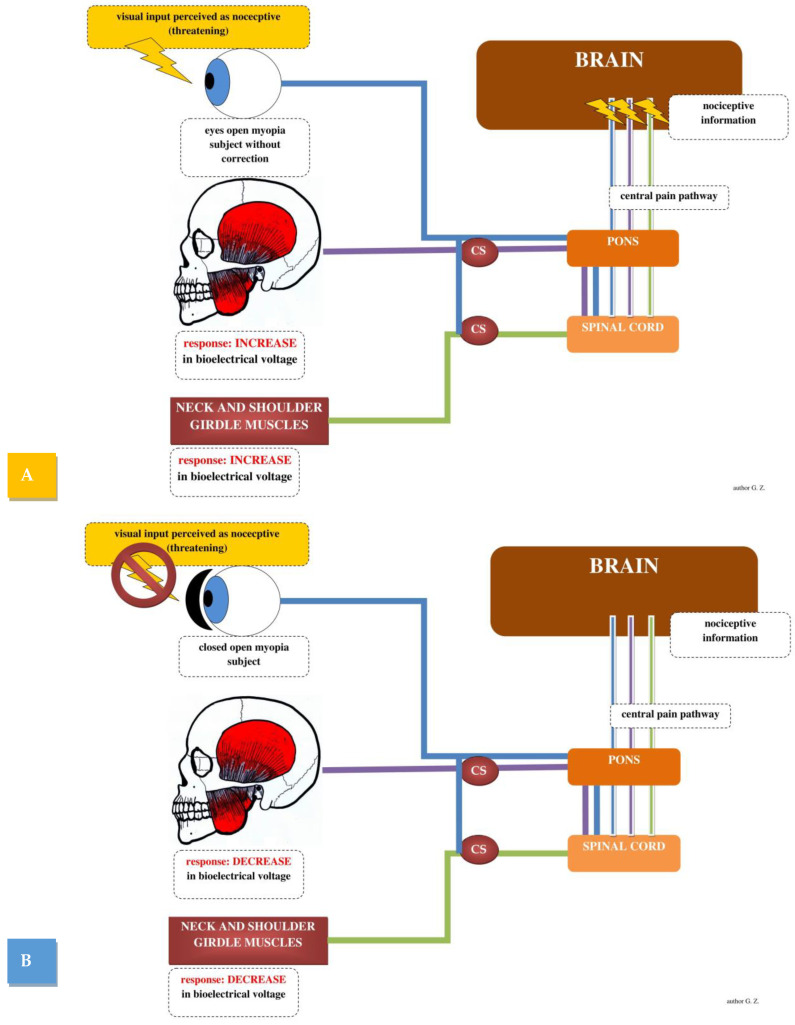
Changes in muscle activity associated with central sensitization (eyes open—(**A**) and eyes closed—(**B**)) in myopic subjects.

A human study indicates that markers of subcortical nociceptive processing at the level of the brainstem were markedly suppressed by accompanying touch; also, studies indicate that touch also inhibited the arrival of ascending nociceptive stimuli to the cortex [60]. These findings indicate that touch inhibits concurrent nociceptive stimuli at the subcortical level, consistent with the results of spinal animal experiments [61,62]. The effect of direct touch on changes in the corneal nociceptor area remains unexplored. Closing the eyes through the eyelids may have a silencing effect on nociceptors within the eyes, and this, via CS nerve pathways, could cause silencing of the interneurons of the efferent muscles of the masticatory organ (Figure 2). The closing of the eyes may be associated with silencing of the nociceptive response, which is located in a different region, e.g., the cervical region and stomatognathic system.

Patients often compensate for vision problems by leaning forward or turning their head from side to side. These individuals often have a protracted position of the head and cervical spine, which leads to increased tension of the thoracic muscles, descending fibers of the quadriceps, scapular lever, sternoclavicular and sternocleidomastoid muscles, and suboccipital muscles [63]. Long-term shortening of the aforementioned muscles can cause ischemia and symptoms including headache and dizziness, tinnitus, and neck stiffness [64]. Long duration of excitation of nociceptors can cause CS in the posterior horn neurons, and open inactive synaptic connections in adjacent segments, leading to an excitatory effect at the spinal cord or brainstem level [65]. Excitation can also be transmitted further through the sensory nuclei of the trigeminal nerve which mediate the impulses of all cranial nerves [57].

The influence of the neck and pectoral girdle muscles on continuous impulsion, and the influence on increased tension in the masticatory muscles have been demonstrated [54]. However, the connections between the cervical spine region, stomatognathic system, and the organ of vision are still not fully explained. Based on the above considerations, CS may explain the changes observed in sEMG in subjects with myopia [32,36], in subjects without visual impairment but with TMDs [30,35], and the lack of response in sEMG recordings in healthy subjects [34].

## 4. Biochemical Connection

Kynurenine (KYN) is a major metabolite of tryptophan (TRP) formed in the kynurenine pathway. The reaction is catalyzed by tryptophan-2,3-dioxygenase (TDO) or indoleamine-2,3-dioxygenases 1 and 2 (IDO). TDO is a constitutive enzyme present mainly in the liver [66]. KYNA is enzymatically produced from a key intermediate compound in the tryptophan catabolic pathway, KYN [67]. KYN and its metabolites, collectively known as kynurenines, exhibit diverse biological activities [66]. Initially, both quinolinic acid and KYNA, a glutamate agonist and antagonist, respectively, were shown to be involved in the control of neurotransmission, neuronal cell membrane excitability, and neurodegeneration [66,68]. It has been shown that KYNA can protect neurons from excitotoxic damage caused by quinolinic acid [69,70]. The retina is the layer of photoreceptor cells and glial cells in the eye that processes light energy, and transmits it to the visual cortex, whose job is to perceive and integrate visual impressions. Damage within the retina can cause loss within the vision [14]. The retina is located in the posterior segment of the eye bulb. It is the innermost layer of the eye localized under other major layers of the eye, which include the choroid and sclera [71]. The kynurenine pathway has been shown to play a role in retinal ontogenesis [72]. Also, the presence of kynurenine and kynurenine pathway enzymes have been demonstrated in the retinas of several vertebrate species [73,74,75]. Increased IDO expression in the retina has been shown in diabetic retinopathy as a result of microglial activation [76]. Recent studies note retinal damage are associated with imbalances in the kynurenine pathway in the damaged retina [77]. The response of the kynurenine pathway to retinal/visual nerve injury depends on the nature of the injury. In particular, mechanical injury to the optic nerve causes a significant decrease in retinal KYNA levels, and this response may be explained by differences in the expression of kynurenine pathway enzymes [77]

Myopic individuals have observable changes in the retina [78,79] and optic nerve [80]. To the best of our knowledge, there is lack of studies analyzing changes in the kynurenine pathway directly in subjects with myopia relative to healthy subjects. Based on the studies cited above, we can hypothesize that changes are present in the kynurenine pathway in the retina and optic nerve in myopic subjects. The role of kynurenines, however, in skeletal muscle is less well understood, and remains largely unknown. It was only recently discovered that there is an intense conversion of KYN to KYNA in exercising skeletal muscles [81,82,83]. It is now broadly believed that physical activity may have beneficial outcomes on the central nervous system, as a result of reducing the amount of circulating KYN available for further metabolism in the brain [84,85]. Based on numerous studies, the link between exercise-induced changes in peripheral kynurenine homeostasis and brain function appears to be indisputable [66,85,86,87]. It has also been demonstrated that skeletal muscle is a heterogeneous tissue composed of cell types with different metabolic profiles, including KYN metabolism, which has implications for KYN-modulating mechanisms [88]. Changes in the metabolism of the kynurenine pathway are evidenced by scientific studies [81,85,88]. The association between physical activity and lower myopia has been recognized in the literature [89]. Also, in temporomandibular disorders, such as myalgia, there was a negative correlation between TRP and pain intensity, and a positive correlation between KYN/TRP and pain intensity [90]. This supports theories of the effect of the kynurenine pathway in myopia.

According to a recent study by Tuka et al. (2021), there is a widespread metabolic imbalance in migraineurs that manifests itself in a completely reduced peripheral catabolism of TRP during the interictal period. It may act as a trigger for migraine attacks, contributing to excess glutamate, induced neurotoxicity, and generalized hyperactivity. This may draw attention to the clinical significance of the kynurenine pathway in migraine [91]. According to studies, KYNA and its derivatives may act as modulators at different levels of the migraine pathomechanism. They can produce antinociceptive effects in the periphery, in the caudate trigeminal nucleus, and can interact with migraine triggers and depression, spreading through the cortex. Experimental data suggest that KYNA or its derivatives may offer a novel approach to migraine therapy [92].

It has been proven that people with refractive errors were more likely to experience migraine episodes [93]. The work of Lajmi et al. (2021) also confirms that abnormal ametropia may be a possible cause of headaches [94]. Research on TMDs versus headache shows that TMDs were associated with an increased incidence of primary headaches. Migraine was the most common primary diagnosis of primary headaches in individuals with TMDs [95]. The association between TMDs and migraines was also noted in the work of Goncalves et al. They demonstrated that in women with TMDs and migraine, migraine improved significantly only when both conditions were treated [96]. The connections discussed between headaches, TMDs, and refractive errors and changes in the kynurenine pathway are purely hypothetical. To our knowledge, there have been no studies on this topic. This needs to be checked. However, the evidence for inter-system connections is quite clear.

**Figure 3:**
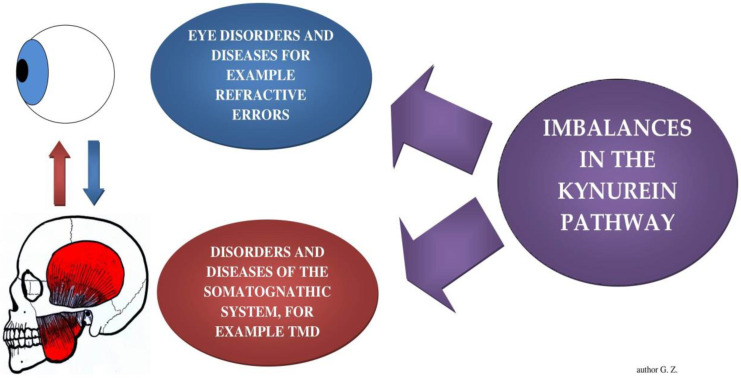
A hypothetical explanation of structural changes in the visual and stomatognathic systems with the kynurenine pathway.

The biochemical linkages discussed above may explain structural changes in individuals with ocular pathologies (ocular convergence defects vs. lateral mandibular deviation [39], astigmatism vs. crossbite [40], myopia vs. Class II division 1 malocclusion [41]). Bollero et al. (2017) noted ocular motor defects versus crossbite and midline deviation [42], and that there is a correlation between TMDs occurrence and visual impairment prevalence [43] (Figure 3).

## 5. Muscular–Tendinous Connection

Traditionally, the word fascia was mainly used by surgeons to describe the stratified tissue surrounding other organs, muscles, and bones [97]. Recently, the definition has been expanded to include all collagen-based soft tissues of the body, including cells that form and maintain the extracellular matrix. The new definition also includes some tendons, ligaments, bursae, endomysium, perimysium, and epimysium [98]. The fascial tissue forms a continuous, three-dimensional, viscoelastic matrix that supports the structure of the entire body. Fascia can be classified as superficial, deep, visceral, or parietal, and further classified according to anatomical location [99]. The classical concept of its passive role in force transmission has recently been refuted [100]. Fascial tissue contains contractile elements that allow a modulating role in force transmission and force generation. The last function not only acts as a transporter of the generated mechanical voltage, but stores mechanical energy to conserve myogenic energy [100,101]. The fascial continuum enables the correct distribution of tension information produced by the various tissues covered or supported by the fascia, allowing the entire body system to interact in real-time [101].

Deep fascia surrounds structures such as bones, muscles, nerves, and blood vessels. It usually has a more fibrous texture, and is rich in hyaluronan compared to the other subtypes. The deep fascia is highly vascularized and contains well-developed lymphatic channels. In some cases, the deep fascia may even contain free motorized nerve endings, such as the corpuscles of Ruffini and Pacini [97]. The deep fascia is a membrane that extends throughout the body, and is held under basal tension by numerous muscle extensions [102]. Based on this anatomical relationship, fascia can transmit the tension generated by muscle contraction to adjacent areas, resulting in stimulation of proprioceptors in that area [103].

The connection between the organ of vision and the muscles of the masticatory organ takes place through the deep fascia of the orbit (Tenon’s fascia) connecting with the deep fascia of the skull (cranial fascia), and through it with the temporal fascia, and then connecting with the deep fascia of the neck [103,104]. Tenon’s fascia is the fibrous layer surrounding the eyeball from the edge of the ciliary body to the entrance of the optic nerve. In the middle part, it attaches to the back of the conjunctiva of the eye, and in the middle part, it connects with the eye muscles—it forms sheaths of four rectus and two oblique muscles, and also connects with the fatty body of the orbit, and forms a continuity with the sheath of the optic nerve [103]. The temporal fascia is a dense fibrous layer covering the temporal muscle, and its surface provides an attachment site for the superficial fibers of the temporal muscle, and through the deep cervical fascia, it connects to the quadrilateral and sternocleidomastoid muscles [103,104].

The discussed anatomy of the fascia, its biomechanics in the transmission of tension, and, in particular, its ability to change the body system in real-time [101], may explain the relationship between the organ of vision and the muscles of the masticatory organ observed during surface electromyography tests with the eyes open and closed.

One of the hypotheses explaining the changes present in myopia may be the alterations in the area of the eyelid network associated with the defect: elongation of the eyeball, which is one of the causes of myopia [105]; and protracted positioning of the head and the cervical spine [63,106]. When the eyes are open, myopia patients may aggravate the increased tension within the muscular–fascial system by visually tonic tension of cervical muscles, main muscles of the masticatory organ, and extraocular muscles. The fascial hypothesis can explain structural changes within systems, as well as changes in bioelectrical tensions, due to the ability of the fascia to respond in real-time.

## 6. Conclusions

Based on the presented review, the nervous, biochemical, and fascial connections between the organ of vision and the stomatognathic system seem undeniable. Understanding the anatomical, physiological, and biochemical interrelationships may allow us to explain the interactions between systems. According to our current knowledge, it is not possible to indicate the main impact pathway, and we think it may be a combination of several presented pathways. Dentists, ophthalmologists, physiotherapists, and optometrists should be increasingly aware of this relationship for better diagnosis and treatment of patients.

## Data Availability

Not applicable.
